# Supplementary material for: A Holistic Approach from Systems Biology Reveals the Direct Influence of the Quorum-Sensing Phenomenon on Pseudomonas aeruginosa Metabolism to Pyoverdine Biosynthesis
Source: Metabolites. 2023 May 16;13(5):659. doi: 10.3390/metabo13050659 (PMC10224149; doi:10.3390/metabo13050659)
Supplement: Supplementary file 1 [file metabolites-13-00659-s001.zip › metabolites-2326047-supplementary file.pdf]

Article

A Holistic Approach from Systems Biology Reveals the Direct Influence of the Quorum-Sensing Phenomenon on *Pseudomonas aeruginosa* Metabolism to Pyoverdine Biosynthesis

Diana Carolina Clavijo-Buriticá [1,*], Catalina Arévalo-Ferro [1] and Andrés Fernando González Barrios [2]

[1] Grupo de Comunicación y Comunidades Bacterianas, Departamento de Biología, Universidad Nacional de Colombia, Carrera 45 No. 26-85, 111321 Bogotá, Colombia; carevalof@unal.edu.co

[2] Grupo de Diseño de Productos y Procesos (GDPP), Departamento de Ingeniería Química y de Alimentos, Universidad de los Andes, Edificio Mario Laserna, Carrera 1 Este No. 19ª-40, 111711 Bogotá, Colombia; andgonza@uniandes.edu.co

* Correspondence: dcclavijob@unal.edu.co

File S1. Data Descriptor Information

Quorum-Sensing Model for the Pyoverdine Expression in *P. aeruginosa.*

Data set: DOI: 10.17632/2xzzkmnpfx.1 (https://data.mendeley.com/datasets/2xzzkmnpfx).

Data license: CC BY 4.0.

Summary and data description: The Quorum-Sensing Model for the Pyoverdine Expression in *P. aeruginosa* was constructed on the standard System Biology Markup Language (SBML) format and represented using System Biology Graphical Notation (SBGN) on the CellDesigner 4.4. software (XML file). The model consists of 114 species: small molecules, proteins, genes with their mRNAs, and nine complexes (Sheet 1 of the Excel file). Additionally, the QS model has 103 reactions, including DNA transcription, mRNA translation, complex protein formation, inhibition reactions, and molecule–molecule interactions with positive, negative, or unknown influences. Each interaction and reaction in the QS model has the kinetic constant value established in the literature (Sheet 2 of the Excel file). The values of the kinetic constants not reported by the literature were assumed according to the ranges of values of the kinetic constants reported for similar molecules. For the construction of the model, 114 ODEs were generated, and an elemental kinetic based on the Mass Action Law was assumed for all reactions that describe the system. The methods to obtain this dataset are described in the methodology of this paper.

P*. aeruginosa* Genome-scale Metabolic Network - CCBM1146.

Data set: DOI: 10.17632/y9htx3fcjm.1 (https://data.mendeley.com/datasets/y9htx3fcjm).

Data license: CC BY 4.0.

Summary and data description: Metabolic network model of *P. aeruginosa* CCBM1146: an improved version of the IMO1056 model genome-scale metabolic network. The model—CCBM1146—which we propose consists of 1146 genes encoding for 826 enzymes, 136 protein complexes, and transport proteins; it contains 1123 reactions and 880 metabolites. The first sheet of the Excel file contains all data of the metabolic model (description of the model). The second sheet contains data about the metabolites list included in the model. The third sheet contains information on the biomass reaction. Finally, the fourth sheet contains data to perform the Flux Balance Analysis. The methods to obtain this dataset are described in the methodology of this paper.
